# COVID-19 Vaccination Scenarios: A Cost-Effectiveness Analysis for Turkey

**DOI:** 10.3390/vaccines9040399

**Published:** 2021-04-18

**Authors:** Arnold Hagens, Ahmet Çağkan İnkaya, Kasirga Yildirak, Mesut Sancar, Jurjen van der Schans, Aylin Acar Sancar, Serhat Ünal, Maarten Postma, Selen Yeğenoğlu

**Affiliations:** 1 Department of Health Sciences, University Medical Center Groningen, University of Groningen (RUG), 9713 AV Groningen, The Netherlands; j.van.der.schans@rug.nl (J.v.d.S.); m.j.postma@rug.nl (M.P.); 2 Department of Infectious Diseases and Clinical Microbiology, Faculty of Medicine, Hacettepe University, Ankara 06230, Turkey; inkaya@hacettepe.edu.tr (A.Ç.İ.); unalserhat57@gmail.com (S.Ü.); 3 Department of Actuarial Sciences, Faculty of Science, Hacettepe University, Ankara 06800, Turkey; kasirgayildirak@gmail.com; 4 Department of Clinical Pharmacy, Faculty of Pharmacy, Marmara University, Istanbul 34854, Turkey; sancarmesut@yahoo.com; 5 Department of Economics, Econometrics & Finance, Faculty of Economics & Business, University of Groningen, 9747 AE Groningen, The Netherlands; 6 Pharmaceutical Care Unit, Faculty of Pharmacy, Marmara University, Istanbul 34854, Turkey; ecz.aylin@gmail.com; 7 Center of Excellence in Higher Education for Pharmaceutical Care Innovation, Universitas Padjadjaran, Bandung 40132, Indonesia; 8 Department of Pharmacology & Therapy, Universitas Airlangga, Surabaya 60115, Indonesia; 9 Department of Pharmacy Management, Faculty of Pharmacy, Hacettepe University, Ankara 06230, Turkey; selen.yegen@gmail.com

**Keywords:** COVID-19, cost-effectiveness, vaccination, Turkey, dynamic modeling

## Abstract

As of March 2021, COVID-19 has claimed the lives of more than 2.7 million people worldwide. Vaccination has started in most countries around the world. In this study, we estimated the cost-effectiveness of strategies for COVID-19 vaccination for Turkey compared to a baseline in the absence of vaccination and imposed measures by using an enhanced SIRD (Susceptible, Infectious, Recovered, Death) model and various scenarios for the first year after vaccination. The results showed that vaccination is cost-effective from a health care perspective, with an incremental cost-effectiveness ratio (ICER) of 511 USD/QALY and 1045 USD/QALY if vaccine effectiveness on transmission is equal or reduced to only 50% of effectiveness on disease, respectively, at the 90% baseline effectiveness of the vaccine. From a societal perspective, cost savings were estimated for both scenarios. Other results further showed that the minimum required vaccine uptake to be cost-effective would be at least 30%. Sensitivity and scenario analyses, as well as the iso-ICER curves, showed that the results were quite robust and that major changes in cost-effectiveness outcomes cannot be expected. We can conclude that COVID-19 vaccination in Turkey is highly cost-effective or even cost-saving.

## 1. Introduction

The first novel coronavirus infection (SARS-CoV-2) was diagnosed in December 2019, and the World Health Organization (WHO) defined the outbreak as a pandemic on 11 March 2020. Since then, COVID-19 has deeply affected health-care services and claimed the lives of almost 2.7 million people worldwide [1]. The pandemic has overwhelmed health care systems and led to global social, health, and economic crises. The workforce and production rate have decreased due to mitigating measures like social distancing, working from home, quarantine, lockdowns, the closure of schools, and outdoor restrictions [2]. To date, there is no established effective medication available against coronavirus infection besides repurposed drugs that are used without definite evidence supporting their effectiveness and safety [3]. Therapeutic monoclonal antibody options are available through emergency-use authorizations against COVID-19, and, though promising, they are still costly and complex in application [4]. Controlling the spread of infection and getting into a normalized post-pandemic situation can only be achieved if immunity rises, either naturally or via vaccines [5]. Consequently, vaccines seem to be the only short-term route to combat the pandemic, and they should be distributed rapidly, efficiently, and equally. Therefore, the best way to rapidly embark on the most efficient and appropriate way to alleviate the current global crisis is to develop and apply safe and effective vaccines to control COVID-19 and its clinical and socio-economic impacts on a global scale [5,6]. For a few months, various vaccines have been applied globally, and further ones are in development, having reached stages of marketing application or advanced in phase 2 and 3 clinical trials.

As the genetic code of SARS-CoV-2 was unraveled in early 2020, a dense global R&D movement with an unprecedented pace was kicked off to develop vaccines against COVID-19 [7]. Based on the preliminary results of the ongoing clinical trials and real-world data of the (candidate) vaccines, it appears that the vaccines are generally well-tolerated, induce an adequate immune response, protect against (serious) symptomatic disease, and potentially protect against asymptomatic infection and subsequent transmission [8,9,10]. The COVID-19-inactivated vaccine CoronaVac (Sinovac, Beijing, China) has been applied in Turkey. Over 10 million people have been given the first dose of the vaccine so far, and 4.5 million have also received the second dose as well [11], the highest numbers so far in the whole Mediterranean region. Most of those vaccinated are healthcare workers and populations of older adults [12].

COVID-19 vaccine development, licensure, and implementation have taken shape under enormous clinical, political, and economical pressure. Despite the desperate need for superfast tracks, the Food and Drug Administration (FDA) decided not to authorize vaccines that would not meet its standards [13]. Relaxing safety regulations for authorizing any new vaccine potentially threatens the trust in global vaccination programs that has been built-up during the last 75 years [14]. Therefore, in recent months, COVID-19 vaccines have undergone careful assessments for registration and implementation [15], and these will continue to apply to newcomers in 2021. Additionally, the stringent monitoring and continuous evaluation of real-world data apply to vaccines being used in daily practice all over the globe.

Experiences from previous vaccination programs have indicated that immediate planning for vaccination is a prerequisite, with specific attention to all stakeholders such as local communities and healthcare providers [5]. Early planning is even more imperative for a COVID-19 vaccine due to the urgent needs. However, financial sustainability should not be forgotten and should be one of the aspects to consider upfront. Thus, anticipating a continued uptake of COVID-19 vaccines, health technology assessment (HTA) bodies should already be conducted integrated analyses of allocative efficiency, affordability, and sustainability. Therefore, the accurate estimation of COVID-19 vaccination program costs, savings on health-care costs, potential broader impacts, and health impact assessments are needed in the context of HTA cost-effectiveness analyses and the strategic planning of allocating potentially scarce stocks of vaccines [16]. Additionally, when aiming for a fair distribution of vaccines with affordable pricing, individual countries should develop country-specific immunization programs and guidelines for its citizens while relying on solid data, preferably originating both from the countries themselves as well as from integrated global databases [17].

Evaluating the population-level impact of COVID-19 vaccines by mathematical modeling is a critical component of vaccines’ HTA decision making and implementation processes. Country-specific National Immunization Technical Advisory Groups (NITAGs) have varying sets of criteria for advising on vaccines, including safety, efficacy, priority, and increasingly containing cost-effectiveness analysis. Modeling studies in various stages during vaccine development provide crucial information to governments, decision-makers, and manufacturers to anticipate, enable, and monitor an efficient implementation [18]. The WHO Strategic Advisory Group of Experts (SAGE) Working Group on COVID-19 Vaccines encourages modeling studies that are suitable for existing epidemiologic and/or economic data or can be validated with such [19]. Therefore, in this study, we aimed to estimate the cost-effectiveness of strategies for COVID-19 vaccinations for Turkey by using mathematical modelling in various potential scenarios.

## 2. Materials and Methods

### 2.1. Overview

An age-structured deterministic dynamic compartmental model was used to estimate the cost-effectiveness of initial vaccination scenarios, e.g., concerning assumptions on transmission effectiveness. Vaccination scenarios were compared to a base scenario without vaccination (as well as stringent lockdowns) for the entire population of Turkey. The time horizon was limited to the first year after the start of vaccination because of uncertainty on waning vaccine effectiveness, the emergence of new variants, and the desire to be conservative on the benefits of vaccination.

**Figure 1:**
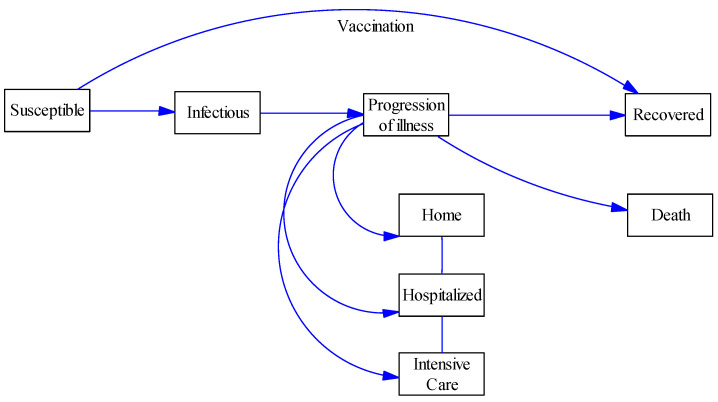
Schematic view of the used compartmental model.

Mathematically, the model was based on a modified SIRD (Susceptible, Infectious, Recovered, Death) compartmental epidemiological model. In such a model, individuals transfer from one compartment to another depending on their health state. In addition to the original SIRD health states, we added a progression of illness states that was subdivided over each location of care for patients before the shift to recovered or death, e.g., home, hospital, and intensive care units. Notably, these locations are critical in relation to the health care costs and health impacts assumed in the model. Figure 1 shows a schematic overview of the used model.

For the base scenario, we assumed a situation in the absence of vaccination and imposed stringent measures, such as lockdowns. Within each vaccination scenario, we varied the uptake level and the effectiveness level of the vaccine. We considered the uptake as the willingness and ability of people to be vaccinated. Effectiveness was considered in terms of effectiveness against disease as well as against transmission, with the latter potentially lower than the former. QALYs (quality-adjusted life years) gained were used as effectiveness indicators, related to healthcare costs, vaccination costs, and productivity losses in the incremental cost-effectiveness ratio (ICER). It can be expected that severe COVID-19 infections may potentially lead to a long-term reduced quality of life, but the extent and duration are highly uncertain. Conservatively, we assumed no long-term impacts beyond the first year of vaccination, except for life-years lost due to COVID deaths, but we did explicitly include QALY impacts in the sensitivity analysis. The willingness-to-pay (WTP) or cost-effectiveness threshold was set at one gross domestic product (GDP) per capita, which can be interpreted as a very cost-effective threshold [20] when using the GDP of 2019 for Turkey: 9127 USD per capita [21]. Additionally, three-times GDP per capita and cost-saving levels were investigated.

### 2.2. Dynamic Transmission Model

The core concept within the dynamic model is the contact matrix reflecting social interactions between age groups underlying potential transmissions within the population. In particular, an age-group specific contact matrix was constructed by using social contact data from a study by Prem et al. [22]. This study estimated the contacts between age groups for 152 countries, including Turkey. Notably, the matrix reflected the number of physical and nonphysical contacts a person has with another person in a specific location (home, work, school, and other locations). However, since this study did not purposely limit contacts at certain location, the overall consolidated contact matrix, which sums the contacts of the different locations, was used. Thus, the differential equation for newly infected and infectious persons per age group (*I_i_*) is as follows:

(1) $$\frac{dI_{i}}{dt}=\beta S_{i}\overset{}{\underset{j}{\sum }}C_{ij}I_{j}/N_{j}-\gamma I_{i}$$

where $C_{ij}$ is the contact matrix over the age classes *i* with *j*, *γ* represents the reciprocal of the infectious period, *N_j_* represents the population of age group *j*, and *β* is the infection rate as defined by the probability of transmission of infection if a contact takes place. Subsequently, the infected persons were distributed over three specific substates, reflecting progression of illness in terms of care locations, i.e., home, hospital, and intensive care unit (ICU). In the model, any still-implemented mild measures (mask-wearing, social distancing, etc.) to control the transmission of COVID-19 were assumed to be uniform over the age groups, compartments, and substates, with vaccination prioritized by age group. An overview of the demographical and epidemiological parameters used can be found in Appendix A.

As COVID-19 does not have a homogenous distribution of the case fatalities over different age groups, the population was divided in four age groups—0–19, 20–39, 40–59, and 60 years and older—aligned with heterogeneity in risk of death from COVID. Specific data of COVID deaths for Turkey were used [23]. Reported case-fatality rates (CFRs) in Turkey only reflect detected COVID-19 deaths and infections, and it could be assumed that these numbers reflect an underreporting of the real infections and deaths. Therefore, the CFRs for each individual age group were estimated using the reported deaths per age group, corrected for excess deaths reported in Turkey in 2020 [23,24,25]. Additionally, the estimated numbers of infections and deaths were calibrated to match the authors’ expert opinions on the likely number of actually infected persons and plausible CFRs. The resulting CFRs appeared similar to estimates by Levin et al. [26].

Quality-adjusted life years lost were estimated by using quality of life data from Turkey [27] (interpolated to fit the modelled age groups) and adjusting the years of life lost according to the 2020 Turkish life tables [28]. Averted QALYs lost were considered as QALYs gained.

### 2.3. Scenarios

Since the number of susceptible and infectious persons at the start of the vaccination was unknown, a run-in period of 360 days prior to the start of vaccination was simulated to estimate these. However, since this estimate is highly uncertain, we subjected these numbers to a thorough sensitivity analysis. Vaccination was assumed to start at day 360 and to coincide with January 2021. A realistic priority in vaccination was assumed, starting with the 60 years and older age group and followed by the younger age groups, with a 60-days interval in between (Turkish Ministry of Health). No distinction between susceptible, infectious, or recovered persons was made for the likelihood to be vaccinated. Additionally, it was assumed that at day 480 (when vaccination is roughly halfway), the social-distancing measures will slowly be reduced in the following 120 days, resulting in a linear increase in the basic reproduction rate R from 1.08 to 2.8, which is the natural reproduction rate in the absence of measures [29]. This assumption was made because populations and governments likely will change behavior and measures after knowing people are vaccinated and the risks of infection declines. Once vaccination is completed on day 600 in the model, the R was linearly increased to 2.8. Vaccination was modelled as an immediate shift of persons from susceptible (*S*) to recovered (*R*). For the vaccination period, the following differential equations were used.

(2) $$\frac{dS_{i}}{dt}=\left(-\beta S_{i}\overset{}{\underset{j}{\sum }}C_{ij}I_{j}/N_{j}\right)-V_{i}$$

(3) $$\frac{dR_{i}}{\begin{array}{c} dt \end{array}}=f_{i}\left(FHm_{i}\;(Hm_{i}\right)+FIc_{i}\;(Ic_{i})+FHs_{i}(Hs_{i}))+V_{i}$$

where $V_{i}$ is the number of people vaccinated; *FHm*, *Fic*, and *FHs* reflect the reciprocals of the durations of stay at home, the ICU, and hospital if ill, respectively; *Hm*, *Ic*, and Hs reflect numbers of ill persons at home, in the ICU, and hospital, respectively; and f is the recovery rate.

To calculate the ICER, a baseline was established where no vaccination was performed, and no lockdown measures were imposed as a comparator. The R_0_ for the baseline was set at 1.8, assuming that individuals will still act differently with the danger of being infected because they will be psychologically and physically influenced by still-occurring social-distancing. This could refer to physical distancing, face masks, using sanitizers, or other (no- or low-cost) careful behavioral actions. Confronting this baseline, two types of vaccination scenarios were compared. The first scenario assumed that vaccine effectiveness on transmission equals effectiveness on disease. The second scenario assumed that vaccine transmission effectiveness is only 50% of effectiveness on disease. Disease effectiveness was defined as the reduced risk a person becoming symptomatically ill and potentially dying. Notably, data on this type of effectiveness are known from clinical trials [10,30]. Transmission effectiveness was defined as the reduced probability that a vaccinated person can still transmit the infection, a subject for which data from RCTs are still scarce and only preliminary real-world data exist [10]. Additionally, data on re-infection after natural infection and infection after vaccination are still scarce. A study concerning COVID-19 re-infections estimated the probability at 0.01% [31]. In the simulations, it was assumed that people once infected with COVID-19 will not be reinfected and are considered immune to COVID-19 for at least the one-year period of our analysis.

Clinical trials of several vaccines showed around 90% vaccine effectiveness on disease [32,33,34,35,36], a figure that was used in this study. Because an average of around 30% of the Turkish population were found to be unsure of getting the vaccine [37], a 70% uptake was used as the base for the vaccination scenarios and numerical comparisons. For the analyses, ICERs for various vaccine effectiveness and uptake combinations, ranging from 30% to 90%, were simulated. These were then tagged as being cost-effective or not. For presentation, iso-ICER curves were constructed to analyze the cut-off points between non cost-effective and cost-effective combinations.

### 2.4. Costs

Costs were split into direct costs and indirect costs. Direct costs included the health care costs of hospitalization, the ICU stay, and pharmacotheraphy at home and vaccination. Indirect costs included production losses due to sickness leave and premature death.

To calculate the direct healthcare costs, unit cost estimates were linked to estimated numbers of persons staying at home, the hospital, or the ICU. In the absence of specific Turkish data, the duration of illness was estimated from international published data at 7.4 days for standard hospitalizations and 17.7 days for the ICU [38,39]. These data were validated with a modest number of hospitalizations in the Hacettepe University Hospital that were available for analysis, suggesting that these estimates were in line. As many persons may have very mild symptoms, in the sheer absence of data, it was conservatively assumed that home recovery involved a period of one day only on average. Subsequently, the healthcare costs per day were used to calculate the total healthcare costs.

To calculate the production losses, the recovery period was marked up from the above-mentioned durations to reach the non-productive period. For recovering at home, 10 days of production losses were assumed based on the quarantine period required in Turkey [23]. This was corrected for the 25% (assumed) working from home during quarantine to 7.5 days. For persons recovering in hospital, the non-productive period was assumed to be 12.35 days. This number included 7.4 days in hospital and an assumed 6.6 days recovering at home. Correcting for the last 25% working from home resulted in a nonproductive period of (7.4 + 6.6 × 0.75 =) 12.35 days. For individuals recovering in the ICU, the non-productive period was set at 28 non-productive days, assuming that persons still would need home recovery afterwards and would not be able to work from home at all. The friction cost approach was used for the valuation of production loss of premature death with the assumption that companies in Turkey will be able to find human resource replacement within the employed or unemployed pool of labor [40]. In the absence of Turkish data but aligned with data from Greece, a 0.27 year period was used for the friction period, [41]. Furthermore, the GDP per person for the corresponding age group was used to monetarize these durations. Notably, the GDP per person per age group was calculated using the number of working people and total wages in each age group (Appendix B). This resulted in 886 USD for 0–19; 13,745 for 20–39; 16,706 for 40–59; and 5649 for 60 years and older. The health care costs for staying at home were assumed to be 1 USD per day for various medications, 110 USD per day for standard hospitalization, and 171 USD per day of ICU stay [42]. The vaccine cost was set at 20 USD/person for two doses in the first year [43,44]. Costs and health outcomes were discounted at a rate of 3% per year to correct for time preference.

### 2.5. Sensitivity Analysis

Sensitivity analysis was carried out by assuming +/−10% change in vaccine costs, QALYs lost, ICU cost, hospitalization cost, percentage population being susceptible, friction period, nonproductive period, and CFRs. An additional sensitivity analysis on the discount rate applied rates of 0% and 5%, compared to the base of 3%. An extensive sensitivity analysis was performed on the effectiveness (base at 90%) and final uptake (70%), resulting in the aforementioned iso-ICER curves. Finally, next to the societal perspective applied as our base with both direct and indirect costs being included, the healthcare perspective was investigated in sensitivity analysis but limited to direct healthcare costs only.

All the simulations were carried out in R Studio 1.3.959 by implementing the four age-group compartmental model using the Runge–Kutta 4 method to approximate the solutions of the differential equations (Appendix C). Additional analyses were done using Microsoft Access and Microsoft Excel.

## 3. Results

### 3.1. Initial Situation

Following our 360-days run-in period, Table 1 shows the estimated epidemiological situation at vaccination start with approximately 86.8% of the population still susceptible to COVID-19, almost 13.0% recovered (immune), and 0.1% infectious and ill. COVID-19 was used as the only cause of death. The share of susceptible persons was found to be critical because it affects the progress of the epidemic and the effectiveness of the vaccination. Additionally, the number of infectious persons was found to play a critical role because it rules the spread of transmissions during the period in which the vaccination is taken up and the pandemic is still causing infections and deaths within the susceptible Turkish population.

### 3.2. Equal Effectiveness on Transmission and Disease

#### 3.2.1. Cost-Effectiveness Analysis

**Figure 2:**
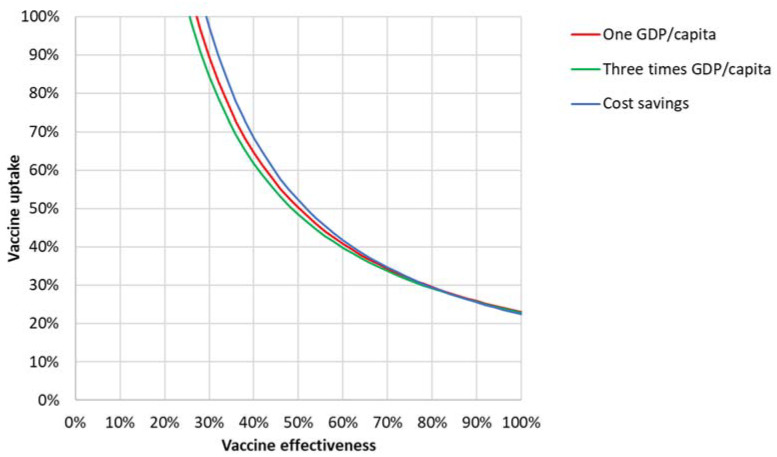
Iso-ICER curves from the societal perspective for one GDP/capita, three-times GDP/capita, and achieving cost savings.

Figure 2 shows the cost-effectiveness results of the various vaccine effectiveness and uptake combinations for the societal perspective, including direct and indirect costs, assuming similar effectiveness on disease and transmission. Three iso-ICER lines are shown: one GDP/capita, three-times GDP/capita, and (if achieving them) cost savings. For example, the figure shows that the 40% effectiveness and 40% uptake combination was not found to be cost-effective because it is located left of the one GDP/capita iso-ICER line. It can also be seen that a vaccine that is over 80% effective was found to be cost-saving at any uptake over 30%. At lower vaccine effectiveness, a higher vaccine uptake would be required to achieve cost-effective or cost-saving situations.

**Figure 3:**
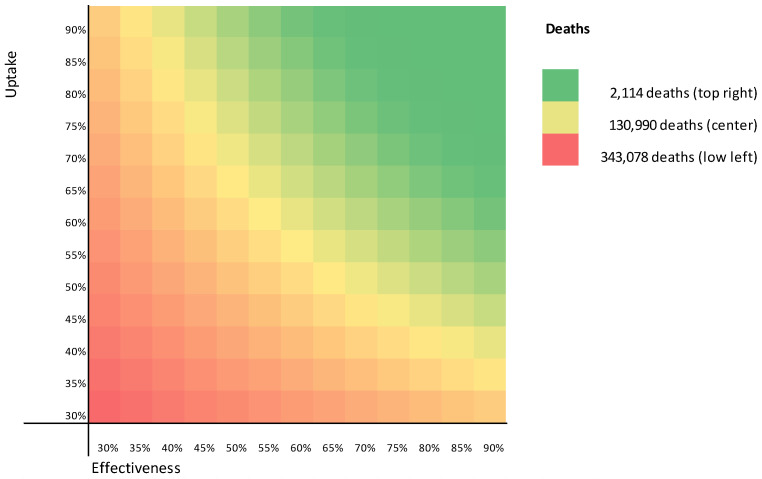
Heatmap of the number of COVID-19 deaths occurring after vaccination.

Figure 3 shows a heatmap of the deaths still occurring in the first year after the vaccination started for different vaccine uptake and effectiveness combinations. As there were still infectious people at the moment vaccination starts and not everybody is vaccinated at once, even a 90% vaccine effectiveness and uptake would still cause 2114 deaths, reflecting the minimum in the heatmap. Central in the heatmap is, e.g., a 60% vaccine effectiveness and uptake causing 130,990 deaths. The maximum number of deaths in the heatmap is 343,078 deaths at a 30% uptake and effectiveness.

**Figure 4:**
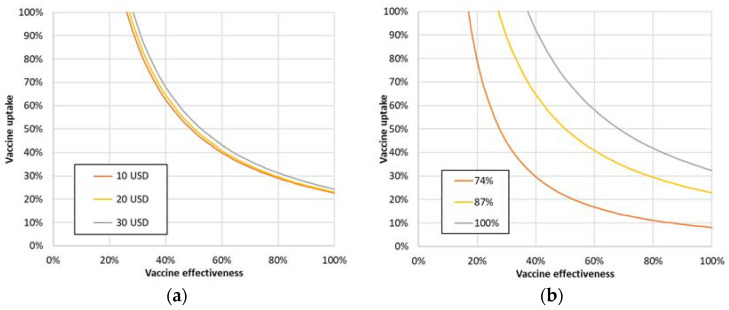
(**a**) Iso-ICER cost-effectiveness curves at one GDP/capita for 10, 20 (baseline), and 30 USD costs of vaccination. (**b**) Iso-ICER cost-effectiveness curves at one GDP/capita for 74%, 87% (baseline), and 100% susceptibility.

Additionally, we varied the vaccination costs. Figure 4a shows that if we assumed a vaccine effectiveness of 90%, the minimum required uptake to be cost-effective was found to be only 25%, 26%, and 27% for vaccine costs of 10, 20, and 30 USD, respectively.

Figure 4b shows that if the share of susceptible persons at vaccination start reduces, more cost-effective combinations of vaccine effectiveness and uptake become available. Using a vaccine effectiveness of 90%, the minimum required uptake to be cost-effective was found to be 37%, 26%, and 9% for 100%, 87%, and 74% susceptible shares, respectively.

#### 3.2.2. Sensitivity Analysis

**Figure 5:**
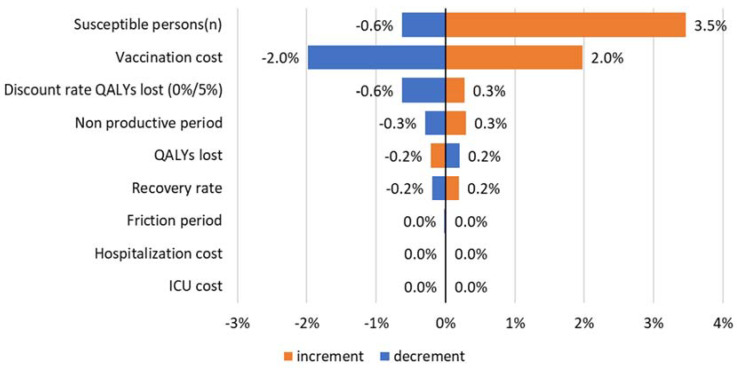
Sensitivity analysis from a societal perspective (+/−10%; except for discount rate QALYs lost at 0% and 5%).

Sensitivity analysis was done with a 90% vaccine effectiveness, a 70% uptake with a vaccine price of 20 USD, and an 87% susceptible population as base. Figure 5 shows the results of the sensitivity analysis.

The number of susceptible persons in the population was found to be the most influential parameter. Vaccination cost was found to affect the ICER with a 2% increase or decrease if price changes by 10%. The other parameters showed even less sensitivity to the results, illustrating its robustness.

### 3.3. Limited Effectiveness on Transmission

#### 3.3.1. Cost-Effectiveness Analysis

**Figure 6:**
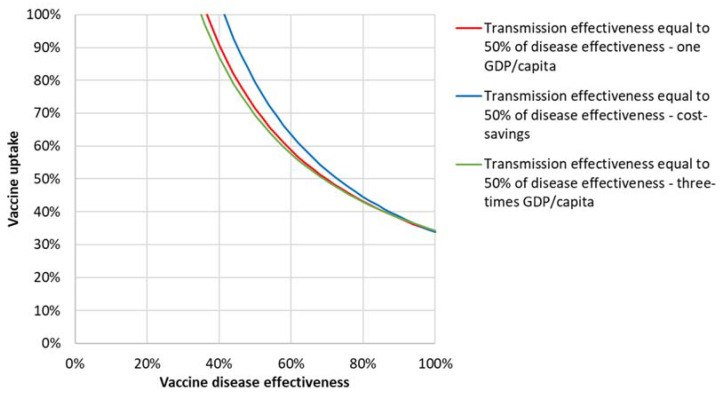
Iso-ICER lines from the societal perspective for transmission effectiveness equal to 50% disease effectiveness for one GDP/capita, three-times GDP/capita, and achieving cost savings.

Figure 6 shows the cost-effectiveness results of vaccine effectiveness and uptake combinations with a limited effectiveness on transmission assumed at 50% of that assumed for disease. Iso-ICER curves are shown at one GDP/capita, three-times GDP/capita, and achieving cost savings.

**Figure 7:**
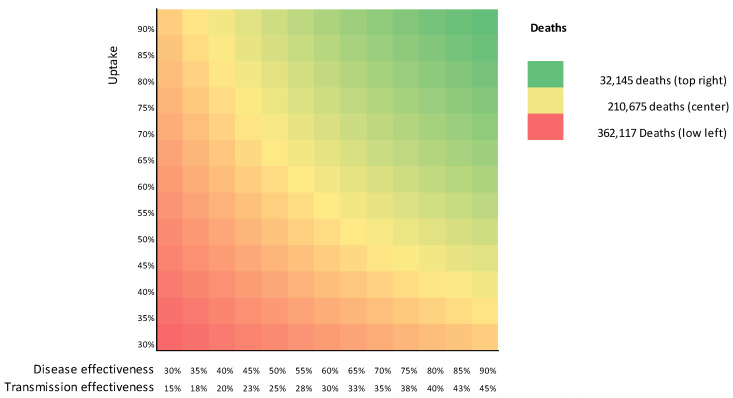
Heatmap of the number of COVID-19 deaths occurring after vaccination starts.

Figure 7 shows a heatmap of the deaths still occurring in the first year after the vaccination started for limited transmission effectiveness. For the scenario with a 90% uptake and disease effectiveness, 32,145 deaths were still estimated to occur—far more than the 2114 for equal transmission and disease effectiveness in Figure 4. Additionally, 60% disease effectiveness and uptake (center) and 30% disease effectiveness and uptake (low left) were found to cause more deaths (210,675 and 362,117, respectively).

#### 3.3.2. Sensitivity Analysis

**Figure 8:**
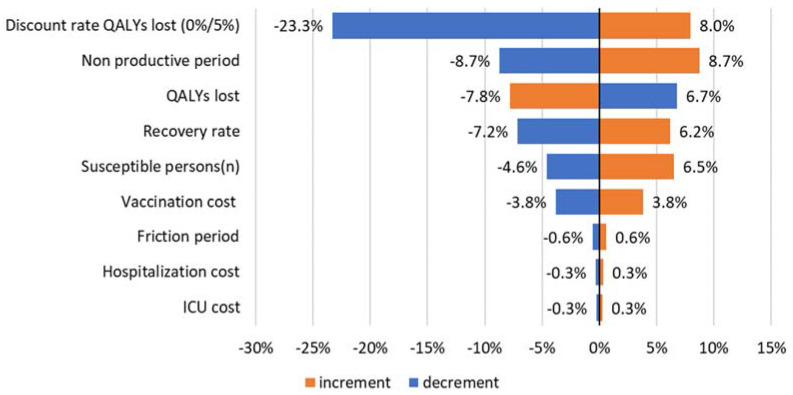
ICER sensitivity analysis (+/−10%; except for discount rate QALYs lost at 0% and 5%) for a limited transmission effectiveness.

A sensitivity analysis for the scenario with limited transmission effectiveness was done using a 90% vaccine disease effectiveness and a 45% transmission effectiveness, a 70% uptake, a vaccine price of 20 USD, and an 87% susceptible population as a baseline. Figure 8 shows the results for the sensitivity analysis.

The discount rate used for the QALYs lost was now found to be the most influential parameter. A 10% change of the non-productive period showed a change of the ICER of +/−8.7%.

### 3.4. Comparison of Scenarios

Table 2 and Table 3 show the summarizing consolidated results for the one-year vaccination period. Together, they comprise a comparison of the two main scenarios: equal effectiveness on transmission and disease and limited effectiveness on transmission. For comparison, the 70% uptake and 90% vaccine disease effectiveness levels, combined with a 20 USD vaccination cost, were used. Table 2 shows that without measures, the number of deaths could reach 211,415, direct health care costs could reach 407,011,036 USD, and indirect cost of production losses could reach up to 6,417,051,139 USD. Both vaccination scenarios obviously improved these numbers.

For equal effectiveness on transmission and disease, 207,421 lives were found to be saved, corresponding to 1,506,501 QALYs gained. To reach this, a direct health care cost of 770,305,902 USD is needed, leading to an ICER from the health care perspective of 511 USD/QALY. Including the indirect cost savings, those were found to lead to overall cost savings. For limited effectiveness on transmission, 122,550 lives, which was equivalent 645,570 QALYs gained, were found to be saved. To reach this, a direct health care cost of 932,279,143 USD is needed, leading to an ICER from the health care perspective of 1045 USD/QALY. Including the indirect cost savings would lead to overall cost savings.

**Figure 9:**
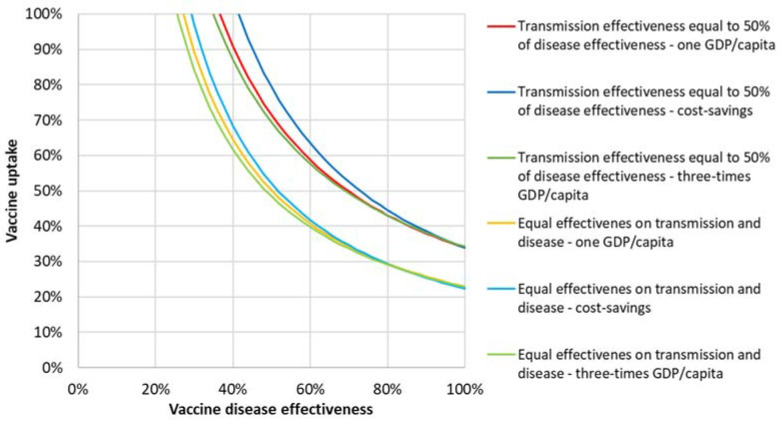
Iso-ICER cost-effectiveness curves at various thresholds for vaccine transmission effectiveness equal to 50% of disease effectiveness and equal effectiveness on transmission and disease.

Figure 9 combines the iso-ICER curves for both vaccination scenarios with similar and limited transmission effectiveness. When we assumed a disease effectiveness of 90%, the minimum required vaccine uptake changed from 27% to 40% if the transmission effectiveness was found to reduce from 100% to 50% of disease effectiveness.

**Figure 10:**
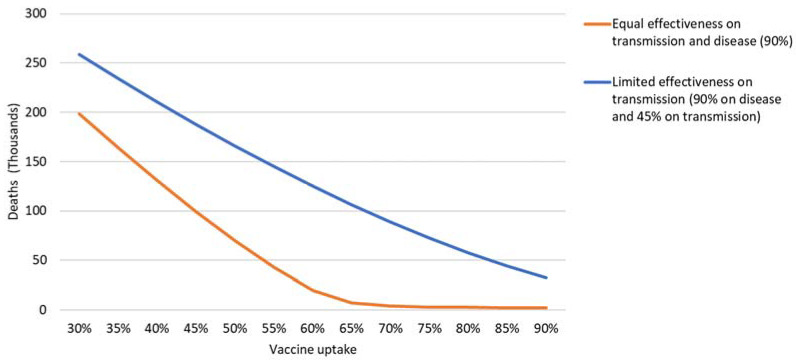
The total number of deaths in the first year after vaccination for the two scenarios for varying vaccine uptakes.

Figure 10 shows the total number of deaths for the first year after vaccination start in the two scenarios for varying uptake rates. For a 90% equal effectiveness on transmission and disease, a clear turning point around 60–65% uptake is shown. This was fully in line with the estimated herd immunity threshold at an R_0_ of 2.8, which can be estimated at 64% by using the standard formula of (R_0_ − 1)/R_0_ [45]. Indeed at 90% effectiveness, 60–65% were found to correspond to 54–59% immune through vaccination—totaling approximately 60–65% again if adding those naturally immune, but not vaccinated—at around 5% at the start of vaccination (40% of 13% immune persons), and approximately 1% of the population were found to be infected during the vaccination. After this turning point, the number of deaths was found to not decrease strongly anymore. This was not the case when we assumed a limited effectiveness on transmission, which led to a linear trend and a similar number of deaths averted with any step taken in uptake increment. Indeed, for this situation, the herd immunity threshold translated into a 100% uptake.

## 4. Discussion

This study analyzed the cost-effectiveness of COVID-19 vaccination in Turkey with a one-year time horizon after the start of vaccination from both the healthcare and societal perspectives. We showed that vaccination with a plausibly assumed effectiveness on disease and transmission of 90% and an uptake of 70% would be cost-effective in Turkey with and an ICER of 511 USD/QALY from the health perspective and even cost saving from the societal perspective. Assuming a halved 45% effectiveness on transmission and still 90% on disease, we found an ICER of 1045 USD/QALY from the health perspective and, again, cost savings from a societal perspective. Therefore, at plausible assumptions for effectiveness and uptake of COVID-19 vaccines in Turkey, vaccination is estimated to be cost-effective or even cost-saving.

Given the various uncertainties, an extensive sensitivity analysis was performed. For example, since our analyses were done in a situation where the vaccination started during an ongoing epidemic, a sensitivity analysis of the level of susceptible persons at vaccination start was performed. It appeared that this parameter was crucial for the model outcomes, and lower levels of susceptible persons at vaccination start lowered the uptake required to achieve a favorable cost-effectiveness. Therefore, increased levels of past natural infections already at vaccination start were found to improve cost-effectiveness. In particular, assuming a 90% vaccine effectiveness led to a reduction of the percentage of susceptible individuals from 87% to 74% and a reduction of the minimum uptake required to be the cost-effective from 26% to 9%.

The real costs of vaccination in the future may change, so a sensitivity analysis of the vaccination cost was performed. Assuming a 90% vaccine effectiveness, the minimum uptake required to be cost-effective was found to barely change from 25% to 27% when the cost of vaccination shifts were varied over a range from 10 to 30 USD. Though the costs/QALY gained increased, this result showed that even an increase of the vaccination costs still justifies vaccination with a relatively low uptake. Obviously, a higher uptake was found to further increase cost-effectiveness up to the herd immunity threshold of around 64%. For limited transmission effectiveness, this changes: the herd immunity threshold at 100% which cannot be achieved in the real world, justifying a vaccination uptake as-high-as-possible.

To adequately present the results of our cost-effectiveness analysis with various varying parameters, the novel concept of iso-ICER curves was introduced. These curves show isolines where the ICER equals a specific value, i.e., one GDP/capita, three-times GDP/capita, and achieving cost savings. The use of iso-ICER curves allowed us to display large numbers of simulation results in a coherent way.

Our results were in line with a study in the USA that found cost savings when prioritizing 65-years-and-older individuals for vaccination from a health care perspective [46]. Economic analyses on vaccination are currently scarce, but there have been some further economic analyses on COVID-19 treatment. Notably, one study performed the cost-effectiveness analysis of Remdesivir for non-ventilated patients and dexamethasone for ventilated patients, ultimately showing cost savings and deaths prevented [47] and therefore supporting favorable economic profiles of these drugs in the treatment of COVID-19. Therefore, treatment likely remains a complementary and potentially cost-effective or cost-saving intervention next to vaccinations. If further treatments that further reduce the fatality rates of infections are developed, the ICERs of vaccination will likely increase. However, because our results were quite robust over large ranges of assumptions and parameter values in scenario and sensitivity analyses, we do not expect that the economic profile of vaccination would drastically worsen.

Our modelling framework integrated international data, e.g., on effectiveness, with country-specific economic, social, and demographic data—in this case, for Turkey. Our model followed the general design of SIRD models and could therefore be seen as conceptually representative for other countries as well. With local data on demography, social contact structures, healthcare costs, GDP, productivity, and wages, our model could also be applied to other countries relatively easily. Indeed, we are currently working on model application for countries in Western Europe, South America, Africa, and Southeast Asia. Methodologically, we want to further develop our model to, e.g., allow for reinfection within the context of newly emerging strains. This might additionally be linked to extending the model’s time horizon and considering waning immunity within a potentially extended time frame.

This study had some limitations. Notably, any model reflects a simplification of reality, and ours specifically assumed various aspects to be homogeneous over all age groups, such as the R_0_, as well as homogeneous mixing between vaccinated and non-vaccinated persons. Additionally, with age-specific contact matrices being the engine of our model, some targeted groups in the population, such as health care workers, could not be specified. Health care workers likely face higher infection rates and potential fatalities and in reality, reflecting a priority group for vaccination [15]. Our model design did not allow for the specific analysis of this vaccination strategy. Additionally, no administration costs related to the vaccination were included, and since recent studies showed antibodies presence six months after vaccination [48], we found it plausible to not include the waning efficacy of immunity (natural and vaccination) in the first year. Both aspects might have worsened cost-effectiveness but could be assumed to be limited and thus have modest influence on the ICER in this study. Conservatively, as we took a time horizon of one year after vaccination start, the impact of QALYs gained due to averted long-term complications of COVID-19, beyond the one-year period, were not included.

A few further assumptions had to be made for the model. In particular, as infections and deaths were likely to be underreported in Turkey, the case fatality per age group was estimated using a modelled number of infections in combination with excess death rates reported in Turkey [25]. These estimates turned out to be slightly lower than reported case fatalities, but they fitted the model logic better. It was estimated that 46,409 persons have so far died in Turkey due to COVID-19. This roughly fit when we applied reported the excess death ratios between 1 and 4 [24,49] in other countries to the reported 28,138 deaths in Turkey [41]. Concerning behavior, it was assumed that people will behave differently if vaccinated, likely mirroring pre-COVID-19 social behaviors. Information on the accuracy of this assumption is absent. We assumed an R_0_ of 1.8 in the absence of vaccination and imposed measures. Though COVID-19 has a natural R_0_ of 2.8 without any restrictions, it was assumed that people will behave differently with continued social distancing measures continuing at low, economically-negligible costs.

In this study, we compared the two vaccination scenarios with a baseline in the absence of vaccinations and imposed measures. However, if we could make a comparison with lockdown impacts included in the alternative baseline, the cost savings of vaccinations would likely further increase. The cost of the lockdown relieve package in Turkey was estimated to cost 4.5% of the GDP [50] or 34.2 billion. Major parts of these costs could be averted with vaccinations. Lockdowns have proven necessary, but they have potentially not been cost-effective. Notably, if we assumed that approximately 160,000 persons’ lives were saved and assumed 7.5 QALYs lost per death (the average from this study), the resulting ICER for lockdowns were found to be 28,500 USD/QALY (=34,200,000,000/(160,000×7.5). Obviously, real-life numbers will be higher than our cost-effectiveness estimates for vaccination.

## 5. Conclusions

We can conclude that COVID-19 vaccination in Turkey is highly cost-effective or even cost-saving compared to a baseline in the absence of vaccination and imposed measures but assuming self-imposed social distancing measures. Our results were found to be robust in an extensive sensitivity and scenario analysis. Finally, if the macro-economic impact of potential lockdowns in the absence of vaccination is considered, the health-economic profile of COVID-19 vaccination in Turkey probably further improves and likely outperforms the economic profile of the lockdowns themselves.

## Figures and Tables

**Table 1:** Estimated epidemiological situation at vaccination start.

Compartment	Persons (n)	%
Susceptibles	72,410,075	86.8%
Infectious and ill	98,217	0.1%
Recovered (Immune)	10,874,915	13.0%
Deaths	46,409	0.1%
Total population	83,429,615	100.0%

Note: Numbers were simulated based on a run-in period of 360 after the first case occurred in Turkey with a total simulated population of 83,429,615, of which 46,409 have been estimated to have so far died due to COVID-19.

**Table 2:** Overview of scenario results from the healthcare and societal perspectives (all costs are in USD, and a discount rate of 3% is used for the QALYs).

Main Scenario	Health Outcomes		Direct Costs		Indirect Costs	
	Deaths	QALYs Lost	Health Care	Vaccination	Sickness Leave	Premature Death
Baseline without vaccination or imposed measures	211,415	1,538,105	407,011,036	-	6,417,051,139	433,671,346
Equal effectiveness on disease and transmission (90% effectiveness)	3994	31,604	9,302,328	1,168,014,610	183,562,183	8,806,634
Limited effectiveness on transmission (90% disease and 45% transmission effectiveness)	88,865	645,570	171,275,569	1,168,014,610	2,676,371,116	182,019,930

**Table 3:** Incremental health outcomes, costs and resulting ICERs for the two vaccination scenarios against the baseline from the healthcare and societal perspectives (all costs are in USD, and a discount rate of 3% was used for the QALYs).

	Incremental Health Outcomes		Incremental Direct Costs	Incremental Indirect Cost Savings	Total Incremental Cost Savings	ICERs	
Scenario	Lives Saved	QALYs Gained				Health Perspective	Societal Perspective
Equal effectiveness on transmission and disease (90%)	207,421	1,506,501	770,305,902	6,658,353,668	5,888,047,767	511	Cost saving
Limited effectiveness on transmission (90% on disease and 45% on transmission)	122,550	892,536	932,279,143	3,992,331,439	3,060,052,296	1045	Cost saving

## Data Availability

The data presented in this study are available on reasonable request from the corresponding author.

## Appendix A. Model Parameters

**Table A1:** Overview of the parameters used in the simulations.

Parameter		Age Classes				General	References
		0–19	20–39	40–59	≥60		
1. General							
	1.1 Total Population					83,429,615	[51]
	1.2 Share of total population (fraction)	0.325	0.310	0.238	0.127		[52]
	1.3 Case-fatality rate (deaths/case)	0.000030	0.000282	0.004858	0.087421		Estimated
	Recovery rate	0.999970	0.999718	0.995142	0.912579		
	1.4 Years of expected life left	64.34	44.92	26.71	8.75		[28]
	1.5 Quality-adjusted years of life left 3% DR	26.54	21.94	14.88	5.40		[27]
	1.6 Quality-adjusted years of life left 0% DR	57.33	38.62	21.02	6.09		[27]
	1.7 Quality-adjusted years of life left 5% DR	18.47	16.34	12.22	5.00		[27]
2. Recovery location fractions after infection							
	2.1 At home	0.999356	0.998336	0.992295	0.875283		[38,39,53] + model
	2.2 Normal hospitalization	0.000628	0.001453	0.005931	0.101664		[38,39,53] + model
	2.3 Intensive care	0.000016	0.000211	0.001774	0.023053		[38,39,53] + model
3. Duration of recovery (days)							
	3.1 At home					1	[38,39]
	3.2 Normal hospitalization					7.4	[38,39]
	3.3 Intensive care					17.7	[38,39]
4. Health care costs per day (USD)							
	4.1 At home					1	Assumption
	4.2 Normal hospitalization					110	[42]
	4.3 Intensive care					171	[42]
5. Productivity costs (USD)							
	5.1 Productivity loss due to premature death	239.30	3711.20	4510.65	1525.29		Calculated
	5.2 Productivity loss due to sickness per day	2.43	37.66	45.77	15.48		Calculated
	5.3 Nonproductive days						
	5.3.1 Home					10	Assumption
	5.3.2 Normal Hospitalization					12.35	Assumption
	5.3.3 Intensive care					28	Assumption
	5.4 Home working share					0.25	Assumption
	5.5 Friction period (year)					0.27	[41]
	5.6 GDP per year per capita	886	13,745	16,706	5649		Appendix B
6. Infectious period						8	[54,55,56,57]
7. Basic reproduction number (R_0_)						2.8	[29]
	7.1 R_0_ in natural measures mode					1.8	Assumption
	7.2 R_0_ in enforced long measures mode					1.08	Assumption
8. Contact matrix—fixed							
	0–19	3.02155	0.91557	0.55907	0.13828		[22]
	20–39	0.95991	1.93832	0.69595	0.12841		[22]
	40–59	0.76151	0.90416	0.72299	0.14798		[22]
	≥60	0.35273	0.31242	0.27712	0.22518		[22]

## Appendix B. GDP per Capita by Age Group

**Table A2:** GDP/capita calculation per age group in Turkey estimated by using the share of the total wage to redivide the GDP by age group.

Age Group	# Persons in Group [52]	# of People Working (2018) [58]	Average Annual Wage (TRY 2018) [58]	Total Wage (TRY)	Share	GDP per Age Group (USD)	GDP/Capita (USD)
0–19	27,087,441	1,580,000	28,117.33	44,425,373,634.55	3%	24,007,496,131	886
20–39	25,836,161	14,066,000	46,718.91	657,148,242,209.70	46%	355,123,268,339	13,745
40–59	19,886,588	11,041,000	55,681.63	614,780,864,598.12	43%	332,227,914,381	16,706
≥60	10,619,425	1,830,000	60,662.68	111,012,695,467.37	8%	59,991,321,150	5649
Total	83,429,615	28,517,000		1,427,367,175,909.73		771,350,000,000	9246

## Appendix C. Differential Equations

Main equations:

(A1) $$N_{i}=S_{i}+I_{i}+R_{i}+Hs_{i}+Ic_{i}+Hm_{i}+D_{i}$$

(A2) $$\frac{dS_{i}}{dt}=\left(-\beta S_{i}\overset{}{\underset{j}{\sum }}C_{ij}I_{j}/N_{j}\right)-V_{i}$$

(A3) $$\frac{dI_{i}}{dt}=\beta S_{i}\overset{}{\underset{j}{\sum }}C_{ij}I_{j}/N_{j}-\gamma I_{i}$$

(A4) $$\frac{dR_{i}}{dt}=f_{i}\left(FHm_{i}\;(Hm_{i}\right)+FIc_{i}\;(Ic_{i})+FHs_{i}(Hs_{i}))+V_{i}$$

(A5) $$\frac{dD_{i}}{dt}=\left(1-f_{i}\right)\left(FHm_{i}\;(Hm_{i}\right)+FIc_{i}\;(Ic_{i})+FHs_{i}(Hs_{i}))$$

Progression of illness state locations:

(A6) $$\frac{dHm_{i}}{dt}=QHm_{i}\left(I_{i}\right)\gamma -Hm_{i}\;\left(FHm_{i}\right)$$

(A7) $$\frac{dIc_{i}}{dt}=QIc_{i}(I_{i})\gamma -Ic_{i}\;\left(FIc_{i}\right)$$

(A8) $$\frac{dHs_{i}}{dt}=QHs_{i}\left(I_{i}\right)\gamma -Hs_{i}\;\left(FHs_{i}\right)$$

Health care costs (excluding vaccination costs):

(A9) $$\frac{dCo_{i}}{dt}=Hm_{i}\left(CHm\right)+Ic_{i}\left(CIc\right)+Hs_{i}\left(CHs\right)$$

Quality-adjusted years of life left:

(A10) $$\frac{dQYLL_{i}}{dt}=\frac{dD_{i}}{dt}QLexp_{i}$$

Productivity loss (due to premature death and sickness days:

(A11) $$\frac{dPLD_{i}}{dt}=\frac{dD_{i}}{dt}CPLd_{i}$$

(A12) $$\frac{dPL_{i}}{dt}=Hm_{i}\frac{PLHm}{FtHm_{i}}CPLr_{i}+Hs_{i}\frac{PLHs}{FtHs_{i}}CPLr_{i}+Ic_{i}\frac{PLic}{Ftic_{i}}CPLr_{i}$$

where:

*f* = recovery rate

*S* = susceptible, *I* = infectious, *R* = recovered, *D* = death

*C* = contact matrix adjusted for non-reciprocity

*N_j_* = total population in group *j*

*R*_0_ = basic reproduction number

*β* = *R*_0_ *γ*/(MaxEigenvalue)

*γ* = 1/infectious period

${FHm}_{i},{FIc}_{i},{\mathrm{and}\;FHs}_{i}=\frac{1}{\mathrm{recovering}\;\mathrm{period}}$

${Hm}_{i}=\mathrm{number}\;\mathrm{of}\;\mathrm{people}\;\mathrm{recovering}\;\mathrm{Home}$

${Ic}_{i}=\mathrm{number}\;\mathrm{of}\;\mathrm{people}\;\mathrm{recovering}\;\mathrm{Intensive}\;\mathrm{Care}$

${Hs}_{i}=\mathrm{number}\;\mathrm{of}\;\mathrm{people}\;\mathrm{recovering}\;\mathrm{in}\;\mathrm{Hospital}$

${CHm}_{i},{\;CIc}_{i},{\;\mathrm{and}\;FHs}_{i}=\cos ts\;\mathrm{per}\;\mathrm{day}\;\mathrm{per}\;\mathrm{location}\;\mathrm{per}\;\mathrm{group}$

${CPLd}_{i}{\;\mathrm{and}\;CPLr}_{i}=\cos ts\;\mathrm{productivity}\;\mathrm{loss}\;\mathrm{in}\;\mathrm{of}\;\mathrm{death}\;\left(d\right)\;\mathrm{and}\;\mathrm{recovered}\;\left(d\right)$
